# A novel temporal identity window generates alternating Eve^+^/Nkx6^+^ motor neuron subtypes in a single progenitor lineage

**DOI:** 10.1186/s13064-020-00146-6

**Published:** 2020-07-28

**Authors:** Austin Seroka, Rita M. Yazejian, Sen-Lin Lai, Chris Q. Doe

**Affiliations:** Howard Hughes Medical Institute, Institute of Neuroscience, University of Oregon, Eugene, OR 97403 USA

**Keywords:** Temporal transcription factor, Motor neuron, Temporal identity, Nkx6, HGTX, Eve

## Abstract

**Background:**

Spatial patterning specifies neural progenitor identity, with further diversity generated by temporal patterning within individual progenitor lineages. In vertebrates, these mechanisms generate “cardinal classes” of neurons that share a transcription factor identity and common morphology. In *Drosophila*, two cardinal classes are Even-skipped (Eve)^+^ motor neurons projecting to dorsal longitudinal muscles, and Nkx6^+^ motor neurons projecting to ventral oblique muscles. Cross-repressive interactions prevent stable double-positive motor neurons. The *Drosophila* neuroblast 7–1 (NB7–1) lineage uses a temporal transcription factor cascade to generate five distinct Eve^+^ motor neurons; the origin and development of Nkx6^+^ motor neurons remains unclear.

**Methods:**

We use a neuroblast specific Gal4 line, sparse labelling and molecular markers to identify an Nkx6^+^ VO motor neuron produced by the NB7–1 lineage. We use lineage analysis to birth-date the VO motor neuron to the Kr^+^ Pdm^+^ neuroblast temporal identity window. We use gain- and loss-of-function strategies to test the role of Kr^+^ Pdm^+^ temporal identity and the Nkx6 transcription factor in specifying VO neuron identity.

**Results:**

Lineage analysis identifies an Nkx6^+^ neuron born from the Kr^+^ Pdm^+^ temporal identity window in the NB7–1 lineage, resulting in alternation of cardinal motor neuron subtypes within this lineage (Eve>Nkx6 > Eve). Co-overexpression of Kr/Pdm generates ectopic VO motor neurons within the NB7–1 lineage – the first evidence that this TTF combination specifies neuronal identity. Moreover, the Kr/Pdm combination promotes Nkx6 expression, which itself is necessary and sufficient for motor neuron targeting to ventral oblique muscles, thereby revealing a molecular specification pathway from temporal patterning to cardinal transcription factor expression to motor neuron target selection.

**Conclusions:**

We show that one neuroblast lineage generates interleaved cardinal motor neurons fates; that the Kr/Pdm TTFs form a novel temporal identity window that promotes expression of Nkx6; and that the Kr/Pdm > Nkx6 pathway is necessary and sufficient to promote VO motor neuron targeting to the correct ventral muscle group.

## Introduction

Neural diversity from flies to mice arises from two major developmental mechanisms. First, neural progenitors acquire a unique and heritable spatial identity based on their position along the rostrocaudal or dorsoventral body axes [1, 2]. Second, temporal patterning based on neuronal birth-order results in individual progenitors producing a diverse array of neurons and glia [1, 3, 4]. Temporal patterning is best characterized in *Drosophila*; neural progenitors (neuroblasts) located in the ventral nerve cord, central brain, and optic lobes all undergo temporal patterning, in which the neuroblast sequentially expresses a cascade of TTFs that specify distinct neuronal identities [3–6]. Although all neuroblasts undergo temporal patterning, the TTFs are different in each region of the brain [3–6]. Similar mechanisms are used in the mammalian cortex, retina, and spinal cord, although many TTFs remain to be identified [7–12].

A major open question is how transient expression of TTFs like Kr and Pdm lead to long-lasting specification of molecular and morphological neuronal diversity. Good candidates for integrating spatial and temporal cues to consolidate motor neuron identity are homeodomain transcription factors expressed in post-mitotic motor neurons [13]. In vertebrates, dorsoventral domains of the spinal cord are partitioned into 12 distinct cardinal classes of neurons – each characterized by development from a common progenitor domain, expression of unique homeodomain transcription factors with cross-repressive interactions to stabilize boundaries, and generating neurons with common morphology [2]. We adapt this nomenclature to define Eve^+^ and Nkx6^+^ (Flybase: HGTX) motor neurons as two “cardinal classes” of motor neurons: each class expresses a homeodomain transcription factor (Eve or Nkx6) with cross-repressive interactions, and each class consists of motor neurons with related neuronal morphology (Eve^+^ motor neurons project to dorsal and lateral longitudinal muscles; Nkx6^+^ motor neurons project to ventral muscle groups) [14].

The *Drosophila* neuroblast 7–1 (NB7–1) is arguably the best characterized system for understanding TTF expression and function. Similar to most other ventral nerve cord neuroblasts, NB7–1 expresses the canonical TTF cascade Hb-Kr-Pdm-Cas with each TTF inherited by the GMCs born during an expression window, and transiently maintained in the two post-mitotic neurons produced by each GMC. The TTF cascade generates diversity among the five Eve^+^ U1-U5 motor neuron progeny of NB7–1: Hb specifies U1 and U2, Kr specifies U3, Pdm specifies U4, and Pdm/Cas together specify U5 [15–18]. Identifying TTF target genes, including transcription factors and cell surface molecules, will provide a comprehensive view of how developmental determinants direct neuronal morphology and synaptic partner choices.

It has long been thought that the cardinal classes of motor neurons derive from distinct progenitors; Eve^+^ motor neurons derive from NB7–1, NB1–1, and NB4–2 whereas Hb9^+^ or Nkx6^+^ motor neurons derive from NB3–1 and others. However, DiI labeling of NB7–1 identified a potentially unknown motor neuron innervating ventral muscles, which is distinct from dorsal and lateral longitudinal muscles targeted by the Eve^+^ motor neurons [19]. The observed ventral projection in this lineage could reflect transient exuberant outgrowths that are lost during larval life, or they could be due to an uncharacterized motor neuron that forms stable synapses with ventral muscles.

Here, we show that a newly discovered Kr/Pdm TTF window generates an Nkx6^+^ Eve^**−**^ motor neuron, born between U3 and U4 in the NB7–1 lineage, that projects to ventral oblique (VO) muscles. We also show that overexpression of Kr/Pdm together, or Nkx6 alone, generates ectopic VO motor neurons based on molecular marker expression. Finally, we demonstrate that Nkx6 is required for proper motor neuron axon targeting to ventral oblique muscles. Our results establish a genetic pathway from TTFs (Kr/Pdm), to a cardinal motor neuron transcription factor (Nkx6) to motor axonal targeting. We also make the unexpected discovery that a single progenitor can alternate production of different cardinal motor neuron classes.

## Results

### The NB7–1 lineage has a Kr^+^ Pdm^+^ temporal identity window that generates an Nkx6^+^ motor neuron

**Fig. 1:**
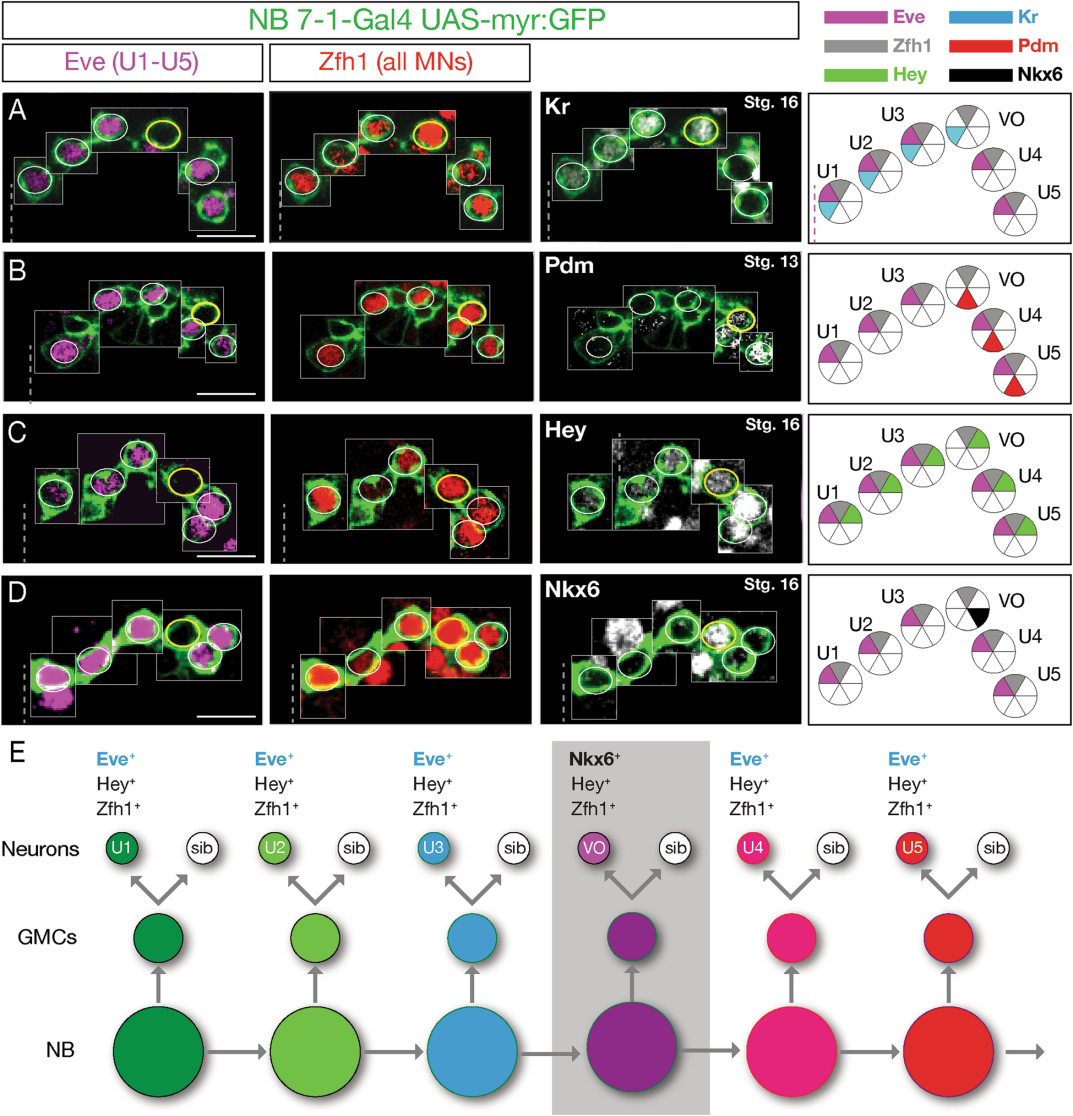
NB7–1 generates an Eve^−^ Nkx6^+^ motor neuron. **a**-**d** The NB7–1 lineage is labeled with GFP (green). One hemisegment of a stage 16 embryo is shown (except where noted); ventral midline, dashed line. Eve^**+**^ Zfh1^**+**^ U1-U5 motor neurons, white circles; Eve^**−**^ Zfh1^**+**^ motor neuron, yellow circle. Neurons are montaged from different z-axis positions with their X-Y position preserved (see Methods). Left column: Eve marks U1-U5 motor neurons. Middle column: Zfh1 marks all motor neurons, although in U2 motor neurons, Zfh1 staining is fainter. Additional Zfh1^**+**^ motor neurons can be seen outside the GFP^**+**^ lineage. Right column: Kr is expressed in U1-U3 and the Eve^**−**^ Zfh1^**+**^ presumptive VO neuron. Pdm is expressed in the Eve^**−**^ Zfh1^**+**^ presumptive VO motor neuron and the Eve^**+**^ U4-U5 motor neurons. Note that Pdm is shown at stage 13 before it fades. Hey is expressed in U1-U5 and the presumptive VO motor neuron, indicating they are all Notch-ON progeny from different GMCs. Note that Hey expression declines after GMC division, resulting in higher Hey levels in the latest born motor neurons (U4/U5). Nkx6 is expressed in the Eve^**−**^ Zfh1^**+**^ presumptive VO motor neuron (yellow circle) and other neurons outside the lineage. Scale bar: 10 μm. Far right column: summary. **e** Proposed NB7–1 lineage

The existence of a Kr^+^ Pdm^+^ temporal identity window had been predicted by computational methods [20], so we sought to confirm this in vivo using a previously-characterized highly-specific NB7–1 split gal4 line (*NB7–1-gal4* [21]) to express *UAS-myr:GFP* in NB7–1 and its progeny (Fig. S1, Fig. 1a-d). This driver line is expressed during the early part of the lineage, including the time U1-U5 neurons are born, but fades out before the end of the lineage [21]. We observed that NB7–1 expresses Kr alone in the early-stage 11 embryo, followed by Kr/Pdm co-expression mid-stage 11, and switches to the sole expression of Pdm by late-stage 11 (Fig. S1). To determine the identity of the neurons originating from the Kr^+^ Pdm^+^ temporal identity window, we used Eve to identify the U1-U5 motor neurons within the lineage, and Zfh1 to label all motor neurons [22]. We identified a single Eve^**−**^ Zfh1^+^ motor neuron in the lineage (Fig. 1a-d). For reasons described below, we call this the VO neuron. This Eve^**−**^ Zfh1^+^ VO motor neuron was Kr^+^ Pdm^+^ (Fig. 1a, b), consistent with originating from the previously defined Kr^+^ Pdm^+^ GMC within the NB7–1 lineage [20].

The U1-U5 motor neurons arise from neurons that have active Notch signaling at their terminal division; their sibling neurons lack active Notch signaling [23]. To determine whether the Kr^+^ Pdm^+^ motor neuron is a U1-U5 sibling, we stained for the Notch reporter gene Hey [24, 25]. As expected, all U1-U5 motor neurons transiently expressed the Notch reporter Hey, leading to strong expression in the latest-born neurons (U4/U5) and weaker expression in the earlier-born neurons (U1-U3). Importantly, Kr^+^ Pdm^+^ motor neuron also expressed Hey (Fig. 1c), and thus the Kr^+^ Pdm^+^ motor neuron is not a U sibling neuron. This further supports our conclusion that it originates from the fourth-born Kr^+^ Pdm^+^ GMC in the NB7–1 lineage [20].

The transcription factors Eve and Nkx6 have cross-repressive interactions [14], raising the possibility that the Kr^+^ Pdm^+^ motor neuron might be Nkx6^+^. Indeed, we confirmed that the Kr^+^ Pdm^+^ motor neuron was Nkx6^+^ (Fig. 1d). Interestingly, this Nkx6^+^ motor neuron was negative for other ventral neuron markers, including Hb9, Islet and Lim3 (data not shown). We conclude that the NB7–1 lineage produces two cardinal classes of motor neurons: Eve^+^ motor neurons and an Nkx6^+^ motor neuron; unexpectedly, these cardinal classes are produced in an alternating mode from a single progenitor lineage: 3 Eve motor neurons > 1 Nkx6 motor neuron > 2 Eve motor neurons (Fig. 1e). This is surprising, and one of the few examples of a progenitor alternating cell types within its lineage (see Discussion).

### The Nkx6^+^ motor neuron projects to ventral oblique muscles

**Fig. 2:**
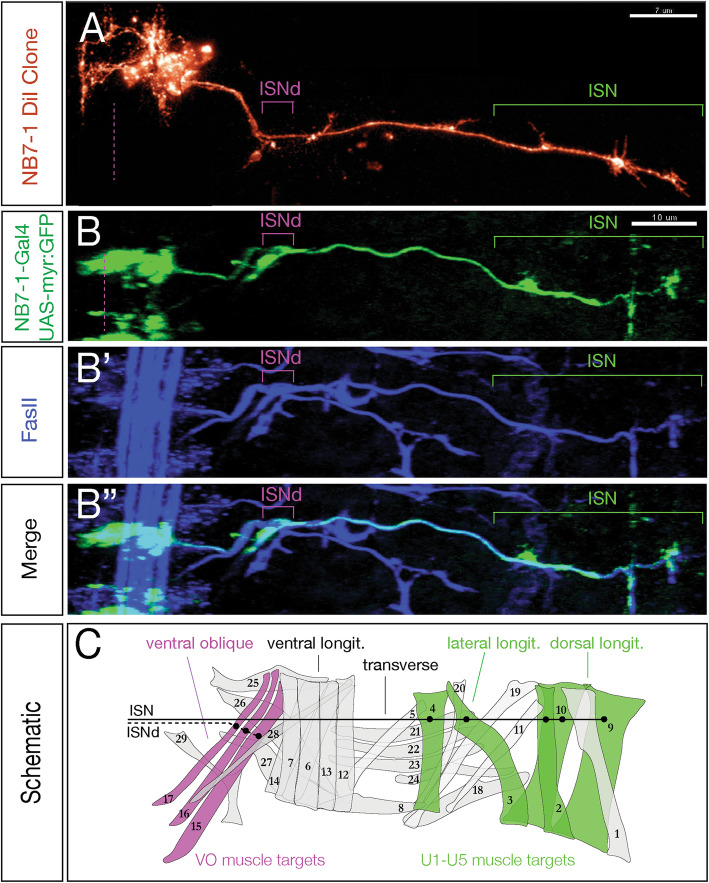
NB7–1 generates a motor neuron that innervates ventral oblique muscles. (A) NB7–1 DiI clone (red) generated as described in Schmid et al. (1999). Green bracket, U1-U5 motor neurons innervating dorsal and lateral longitudinal muscles; magenta bracket, unknown neuron(s) innervating more ventral muscles. Scale bar: 7 μm. (B-B″) NB7–1 lineage marked by GFP (green) stained for the motor axon marker FasII (blue). Stage 17; one hemisegment shown; ventral midline, dashed at left; dorsal to the right. The NB7–1 lineage produces motor neurons branching out to innervate dorsal and lateral longitudinal muscles (green bracket) and ventral muscles (magenta bracket), similar to the DiI clone in panel A. Scale bar: 10 μm. (C) Schematic of muscle groups, including the ventral oblique muscles (magenta) and the dorsal and lateral longitudinal muscles (green). The U1-U5 motor neurons of the NB7–1 lineage project to dorsal and lateral longitudinal muscle targets through the ISN (black line), while an unidentified subset of NB7–1 progeny project to ventral oblique muscle targets through ISNd (dashed line)

All of the Nkx6^+^ motor neurons that have been characterized to date project to ventral body wall muscles [14]. To identify the muscle target of the Kr^+^ Pdm^+^ Nkx6^+^ motor neuron in the NB7–1 lineage, we first examined single neuroblast DiI clones [19] and looked for muscles with innervation distinct from the known U1-U5 dorsal muscle targets. DiI labeling of NB7–1 marked all clonal progeny at embryonic stage 17, and showed innervation of all known U1-U5 dorsal and lateral longitudinal muscle targets, plus innervation of the ventral oblique muscles 15, 16, and 17 via the intersegmental nerve d branch (ISNd) (Fig. 2A). We independently confirmed these results using *NB7–1-gal4* to drive GFP expression, which labeled all U1-U5 dorsal and lateral longitudinal muscle targets plus ventral oblique muscles in a majority of hemisegments (Fig. 2B-C; 54/93 hemisegments. These data are consistent with the Nkx6^**+**^ Eve^**−**^ motor neuron in the NB7–1 lineage projecting to ventral oblique muscles, but without single neuron labeling we can’t make a conclusive match.

**Fig. 3:**
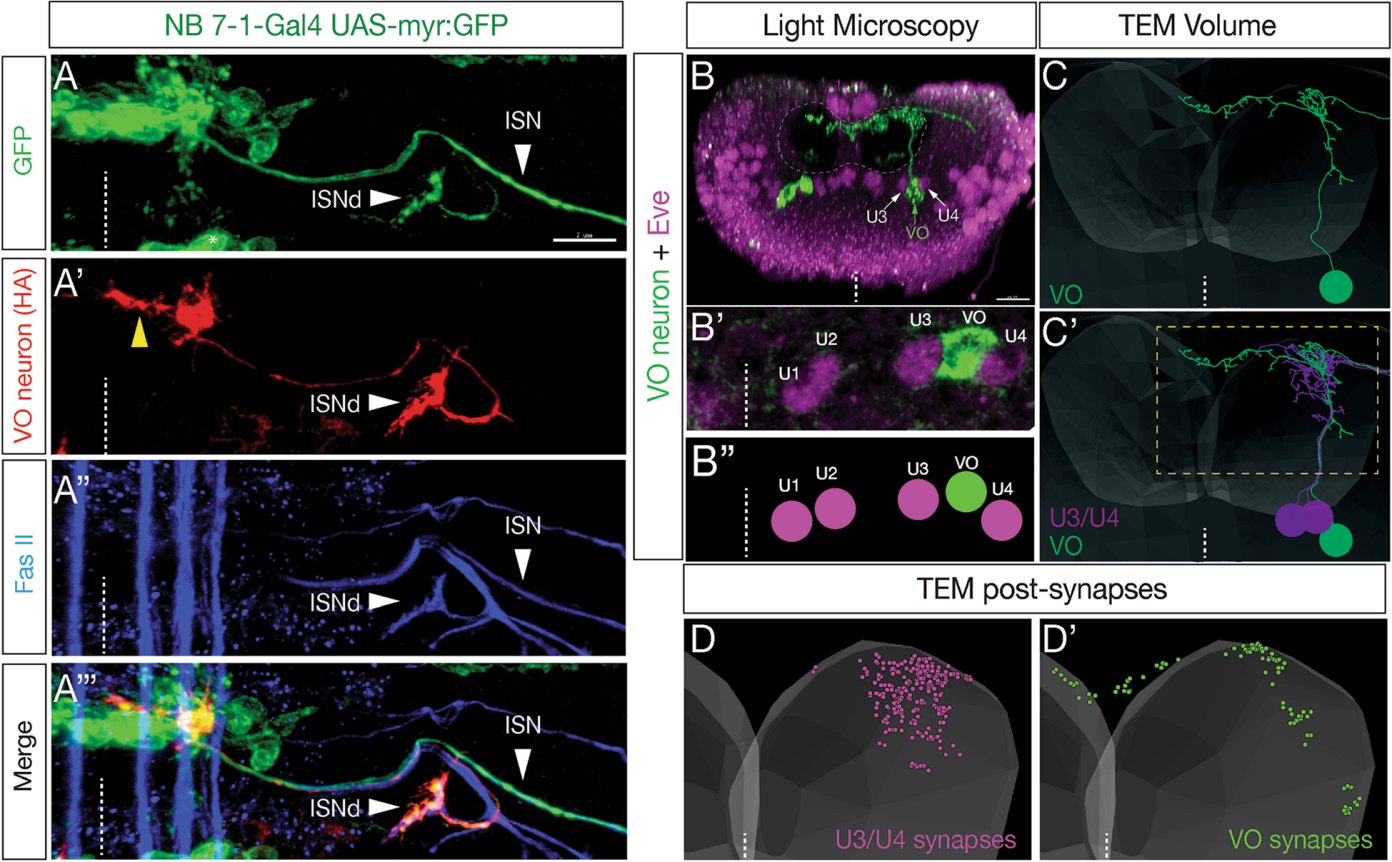
NB7–1 derived Nkx6^+^ motor neuron projects axon to ventral oblique muscles and dendrites to the dorsal neuropil. (A-A"‘) Single cell flip out labels a single VO motor neuron with HA (red) within the NB7–1 lineage (green). Note the single VO neuron has an axon projection to the ISNd nerve branch known to innervate the ventral oblique muscle group, and a dendritic projection to the midline (yellow arrowhead). FasII staining shows the ISN and ISNd nerves (blue). Stage 16 embryo; ventral midline, dashed line. Scale bar: 7 μm. (B-B″) Single cell flip out labels a single Eve^**−**^ VO motor neuron (green) with an axon projection exiting the CNS and its dendrites projecting to the dorsal-most region of the neuropil. Eve^+^ U3/U4 motor neurons, magenta; white arrows in B. Newly hatched larva; posterior (cross-section) view; dorsal up, ventral midline, dashed line; neuropil boundary, dashed circle. VO motor neuron cell body lies between the U3 and U4 neuron cell bodies (white arrows), similar to its position in the late embryo (see Fig. 1). Scale bar: 10 μm. (C,C′) The VO motor neuron (green) is identifiable in a TEM reconstruction of the newly hatched larval CNS, previously named MN15/16/17 [26]. Note that the VO has similar morphology in both light and TEM volumes (compare panels B and C), and that the VO has a similar cell body position between U3/U4 (compare panels B′ and C′). Posterior (cross-section) view; dorsal up, ventral midline, dashed line; approximate neuropil boundary, translucent shading; only neurons in A1R are shown. Dashed box, region enlarged in D,D’. (D,D’) VO and U3/U4 form postsynapses in different regions of the neuropil. Dorsal up, midline, white dashed line; only neurons in A1R are shown

To visualize the morphology of individual motor neurons in the NB7–1 lineage, we generated single cell flip out clones (see methods for genotype) to stochastically label single neurons with HA (Fig. 3A‘). In addition, we used GFP to label all neurons in the lineage and FasII to detect all motor axons and their muscle targets (Fig. 3A-A"’). As expected, full lineage labeling revealed innervation of ventral oblique muscles via ISNd. In addition, HA labeling identified an individual motor neuron that specifically targeted the ventral oblique muscles via ISNd (Fig. 3A’-A"’; Supp. Movie 1). This motor neuron had an ipsilateral dendritic process that approached the midline (Fig. 3A’, yellow arrow), indistinguishable from the previously identified MN17 that innervates the ventral oblique muscles [27]. For this reason, we call the Kr^+^ Pdm^+^ Nkx6^+^ motor neuron in the NB7–1 lineage the VO motor neuron.

We next wanted to determine whether the Nkx6^+^ VO motor neuron and the Eve^+^ U1-U5 motor neurons have distinctive dendritic morphology or premotor innervation, as expected based on their distinctive axon targeting to different muscle groups. We repeated the sparse-HA labeling experiment in newly hatched larvae (Fig. 3B-B") so we could identify VO motor neuron morphology, and identify it in a TEM atlas of all abdominal motor neurons in newly hatched larvae [26]. We identified the NB7-1 VO motor neuron in the TEM volume based on its characteristic central nervous system (CNS) projections and cell body position between U3 and U4 (compare Fig. 3B and C). Interestingly, VO and U3/U4 motor neurons had different dendrite projections (Fig. 3C’), as well as different postsynapse locations within the neuropil (Fig. 3D,D’). Furthermore, they had distinctive premotor inputs: top inputs to VO are A06c and A18b2 interneurons, whereas top inputs to U3/U4 are A31k and A18a interneurons [26]. We conclude that the NB7-1 lineage produces two cardinal classes of motor neurons: dorsal projecting Eve^+^ motor neurons and a ventral projecting Nkx6^+^ VO motor neuron, each with distinct morphology and connectivity.

### Overexpression of Kr/Pdm generates ectopic Nkx6^+^ VO motor neurons

**Fig. 4:**
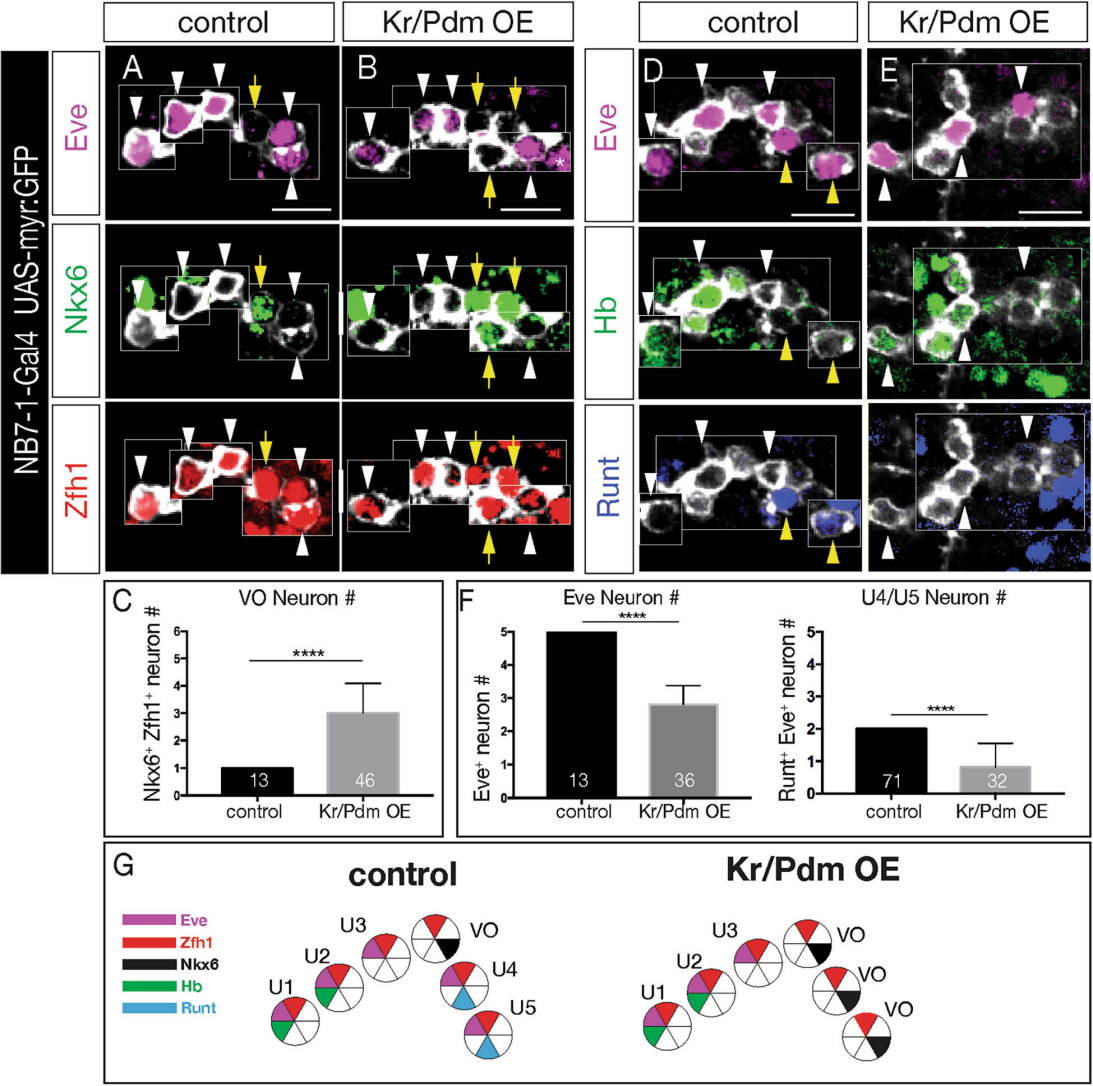
Kr/Pdm co-expression induces ectopic Nkx6^+^ VO motor neuron molecular identity. **a**-**c** Kr/Pdm overexpression (OE) induces ectopic Nkx6^+^ VO motor neurons. (A) Control lineages (*NB7–1-Gal4 UAS-myr:GFP*) have one Nkx6^+^ Eve^**−**^ VO motor neuron (yellow arrow). **b** Kr/Pdm overexpression (*NB7–1-Gal4 UAS-myr:GFP UAS-Kr UAS-Pdm*) increases the number of Nkx6^+^ Eve^**−**^ VO motor neurons. **c** Quantification. Asterisk, GFP-negative EL neurons from NB3–3. Scale bar: 10 μm. **d**-**f** Kr/Pdm overexpression (OE) reduces Eve^+^ motor neurons. **d** Control lineages (*NB7–1-Gal4 UAS-myr:GFP*) have five Eve^+^ motor neurons including Hb^+^ U1/U2 and Runt^+^ U4/U5. **e** Kr/Pdm overexpression (*NB7–1-Gal4 UAS-myr:GFP UAS-Kr UAS-Pdm*) reduces the number of Eve^+^ U4/U5 motor neurons. **f** Quantification. Scale bar: 10 μm. **g** Summary

Hb, Kr, Pdm, and Pdm/Cas each specify a distinct temporal identity within multiple neuroblast lineages [6]. In contrast, the newly discovered Kr/Pdm temporal identity window [20] has not yet been tested for a role in specifying neuronal identity. Here we ask whether co-expression of Kr and Pdm can induce ectopic VO neurons. To test this hypothesis, we overexpressed Kr and Pdm together specifically in the NB7–1 lineage (*NB7–1-gal4 UAS-Kr UAS-Pdm UAS-myr:GFP*). Controls always had an Eve^**−**^ Nkx6^+^ Zfh1^+^ VO motor neuron located between the Eve^+^ U3 and U4 (Fig. 4a; quantified in 4c). In contrast, Kr/Pdm co-expression resulted in 2–3 additional Nkx6^+^ Zfh1^+^ VO motor neurons (Fig. 4b; quantified in 4c). We note that Kr/Pdm overexpression was not able to alter earlier temporal identities (Hb^+^ U1/U2 and Kr^+^ U3 neurons), similar to the well-characterized inability of later TTFs to alter earlier TTF cell fates [15–18, 28]. We conclude that Kr/Pdm TTFs can induce Nkx6^+^ VO motor neuron identity beginning with the fourth division of the NB7–1 lineage. It is unknown if Kr/Pdm acts directly or indirectly to promote *nkx6* expression (see Discussion).

We next asked whether Kr/Pdm co-expression delays production of the later-born Eve^+^ U4-U5 motor neurons until after birth of the ectopic VO motor neurons (lineage extension model), or replaces the Eve^+^ U4-U5 motor neurons with ectopic VO motor neurons (conversion model). Both outcomes have been observed following misexpression of other temporal transcription factors [18, 21, 29, 30]. We overexpressed Kr/Pdm in the NB7–1 lineage (*NB7–1-gal4 UAS-Kr UAS-Pdm UAS-myr:GFP*), and assayed the fate of the Eve^+^ U1-U5 motor neurons. In controls, we always observed five Eve^+^ U1-U5 motor neurons, including two Runt^+^ U4/U5 motor neurons (Fig. 4d; quantified in 4f). In contrast, Kr/Pdm overexpression led to a loss of the Runt^+^ U4/U5 motor neurons (Fig. 4e; quantified in 4f). We conclude that overexpression of Kr/Pdm in the NB7–1 lineage generates ectopic Nkx6^+^ VO motor neurons at the expense of the later-born U4/U5 motor neurons, supporting the conversion model (summarized in Fig. 4g).

### Overexpression of Kr/Pdm generates ectopic Nkx6^+^ motor neurons that project correctly to ventral oblique muscles

**Fig. 5:**
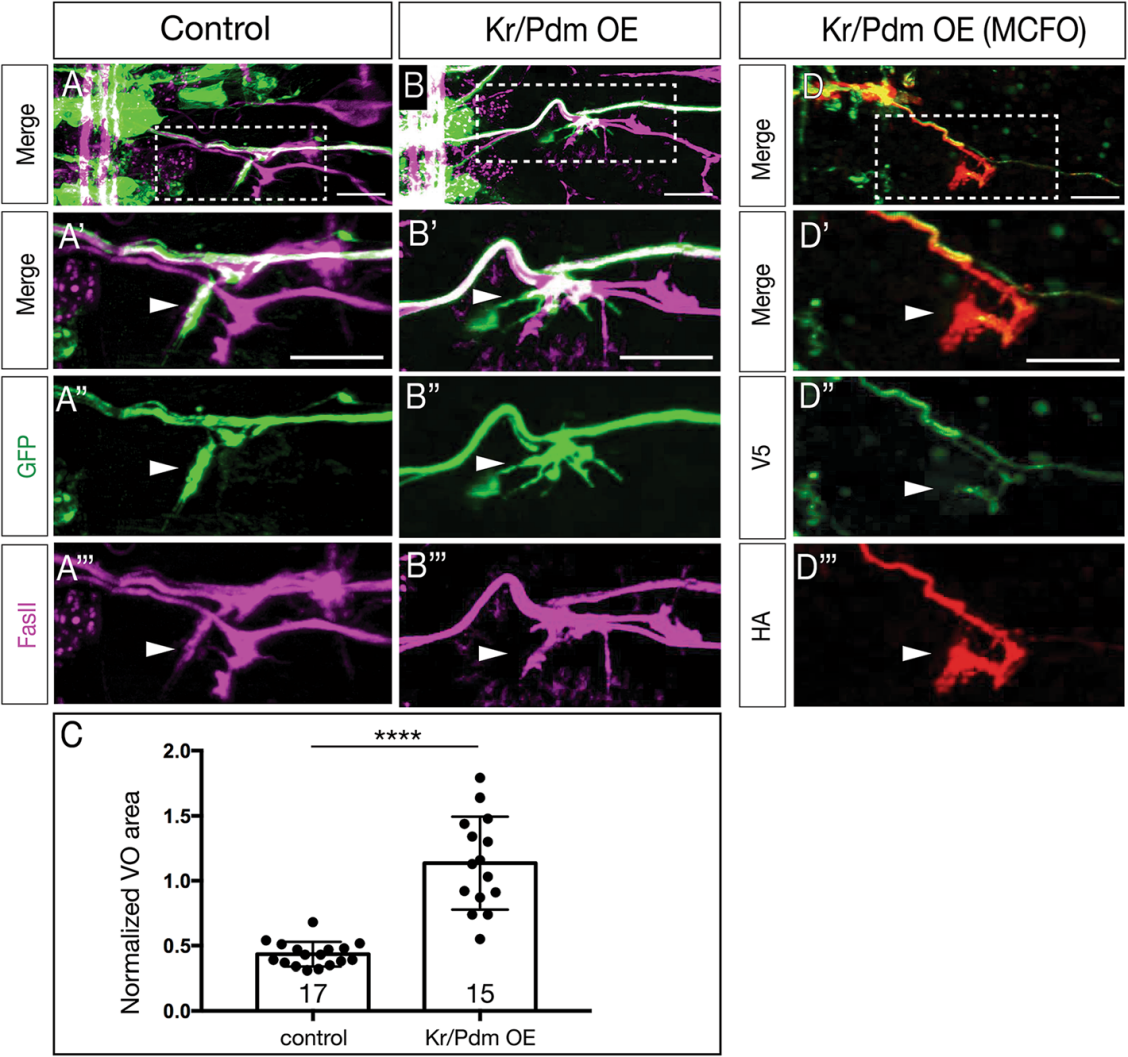
Kr/Pdm induces ectopic motor neurons targeting ventral oblique muscles. (A-C) Kr/Pdm overexpression results in ectopic motor neuron projections to the ventral oblique muscles (arrowhead). (A) Controls (*NB7–1-gal4 UAS-myr:GFP*) show NB7–1 progeny innervating the ventral oblique muscle (ISNd). (A’) Enlargement of boxed region in A. (A") GFP marking NB7–1 progeny; (A"‘) FasII marking all motor axons. (B) Kr/Pdm overexpression (*NB7–1-gal4 UAS-myr:GFP UAS-Kr UAS-Pdm)* induces ectopic VO motor neuron projections to the ventral oblique muscles (arrow). Scale bar: 10 μm. (C) Quantification. See Fig. S2 for methods. (D) Kr/Pdm overexpression analyzed by single neuron MCFO showing two motor neurons projecting to the ventral oblique muscles. (D’) Enlargement of boxed region in D. (D", D"‘) Individual motor neurons labeled with V5 or HA projecting to ventral oblique muscles. Scale bar: 10 μm

In the section above, we show Kr/Pdm overexpression can induce ectopic VO motor neurons based on molecular markers. Here we determine whether Kr/Pdm overexpression can generate ectopic VO motor neurons that project correctly to ventral oblique muscles. We used *NB7–1-gal4* to drive prolonged co-expression of Kr/Pdm, membrane targeted GFP to visualize axon projections, and FasII to identify the ISNd branch to the ventral oblique muscles. In controls, NB7–1 progeny projected in the ISNd to innervate ventral oblique muscles (Fig. 5A, S2, S3; axon volume in ISNd quantified in Fig. 5C; Fig. S2). Following Kr/Pdm overexpression specifically in the NB7–1 lineage, we detected increased axon volume at the ISNd (Fig. 5B, S2, S3; quantified in Fig. 5C), consistent with ectopic VO motor neurons taking the normal VO pathway via ISNd to the ventral oblique muscles. To conclusively show that multiple ectopic VO motor neurons target the ISNd, we used multi-color flip out (MCFO) [31] to express HA and V5 on different neurons within the NB7–1 lineage. Following Kr/Pdm overexpression specifically in the NB7–1 lineage, we identified NB7–1 lineages with distinct HA^+^ and V5^+^ motor neurons that projected via ISNd to the ventral oblique muscles (Fig. 5D). We conclude that prolonged Kr/Pdm co-expression is sufficient to generate VO motor neurons that correctly project out of the ISNd nerve root to innervate ventral oblique muscles. Thus, Kr/Pdm induces both molecularly and morphologically normal VO motor neurons.

### Nkx6 is necessary and sufficient to generate VO motor neurons in the NB7–1 lineage

**Fig. 6:**
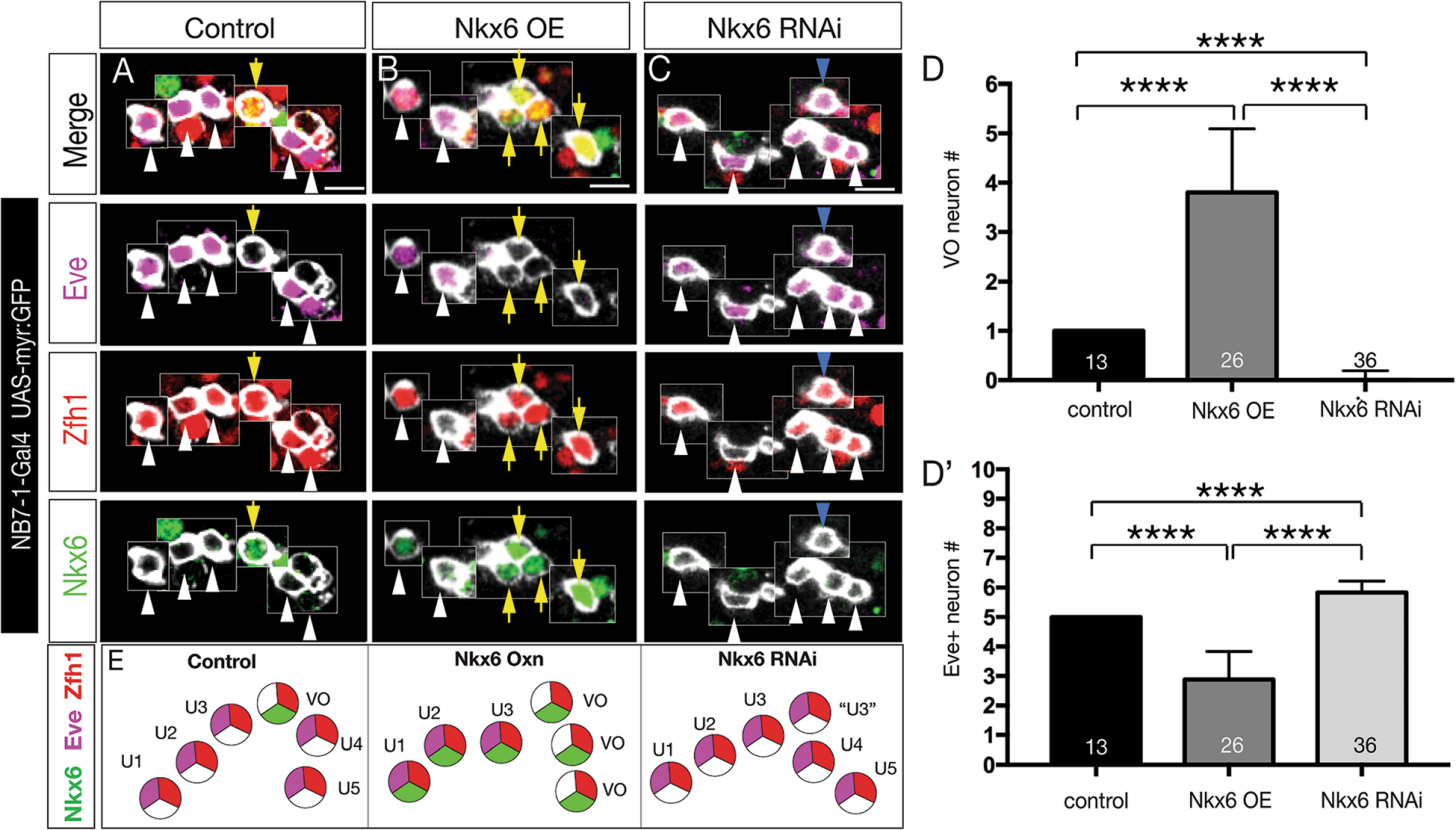
Nkx6 is necessary and sufficient to specify VO motor neuron molecular identity. (A) Controls (*NB7–1-gal4 UAS-myr:GFP*) have one Nkx6^+^ Eve^**−**^ VO motor neuron. (B) Nkx6 overexpression (*NB7–1-gal4 UAS-myr:GFP UAS-Nkx6*) results in ectopic VO motor neurons at the expense of Eve^+^ U3-U5 motor neurons. (C) Nkx6 RNAi (*NB7–1-gal4 UAS-myr:GFP UAS-Nkx6-RNAi*) lack the VO motor neuron and possess an ectopic Eve^+^ “U?” motor neuron at that location (cyan arrowhead). (D, D’) Quantification. (E) Summary. Scale bars, 5 μm

We have shown above that the TTF combination of Kr/Pdm can induce Nkx6^+^ motor neurons that project to the ventral oblique muscles. This raises the question of whether there is a linear genetic pathway from Kr/Pdm to Nkx6 to VO motor neuron identity, or whether Kr/Pdm drives expression of multiple genes required for VO motor neuron identity. Thus, we asked if Nkx6 alone was sufficient to specify VO motor neuron identity. In controls (*NB7–1-gal4 UAS-myr:GFP*), we always detected the U1-U5 Eve^+^ Zfh1^+^ motor neurons and a single Nkx6^+^ Zfh1^+^ VO motor neuron (Fig. 6A). In contrast, overexpression of Nkx6 (*NB7–1-gal4 UAS-myr:GFP UAS-Nkx6*), produced loss of U3-U5 Eve^+^ motor neurons and ectopic VO motor neurons (Fig. 6B; quantified in 6E). The opposite phenotype was observed when we reduced Nkx6 levels by RNAi (*NB7–1-gal4 UAS-myr:GFP UAS-Nkx6-RNAi*): loss of the Nkx6^+^ Zfh1^+^ VO motor neuron and the production of a single ectopic Eve^+^ Zfh1^+^ motor neuron (Fig. 6C; quantified in 6E). We conclude (1) that Nkx6 is necessary to consolidate a VO motor neuron fate from the fourth GMC in the lineage, because without Nkx6 this GMC generates a sixth Eve^+^ neuron; and (2) that Nkx6 is sufficient to specify VO motor neuron identity following production of the fourth GMC in the NB7–1 lineage.

**Fig. 7:**
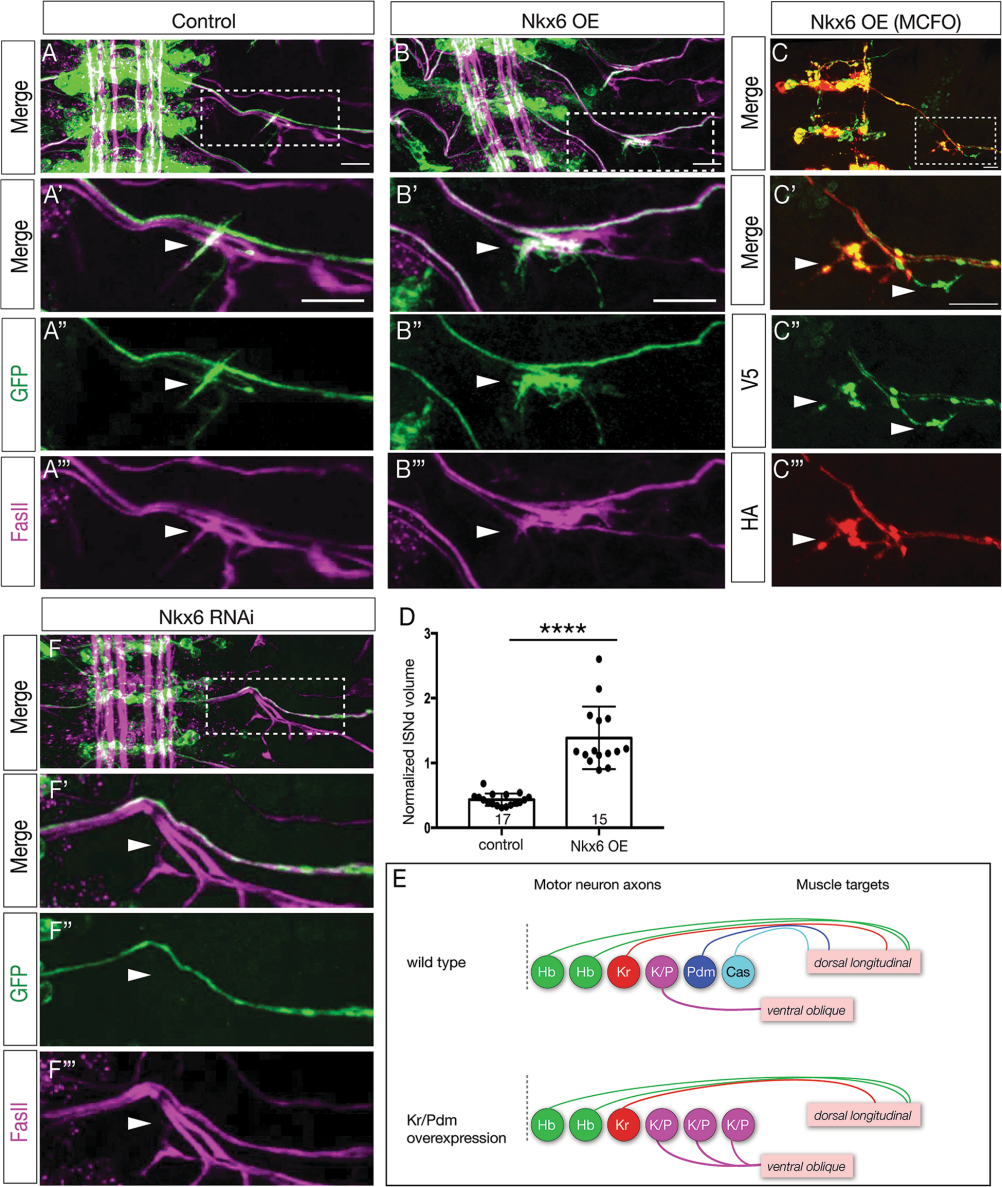
Nkx6 is necessary and sufficient to specify VO motor neuron axon targeting to ventral oblique muscles. **a** In controls (*NB7–1-gal4 UAS-myr:GFP*), NB7–1 progeny project to the ventral oblique muscles. **b** Nkx6 overexpression (*NB7–1-gal4 UAS-myr:GFP UAS-Nkx6*) leads to ectopic projections to ventral oblique muscles. Scale bar: 10 μm. **c** MCFO labeling of single motor neurons in the NB7–1 lineage shows two different HA^+^ and V5^+^ motor neurons projecting via the ISNd to the ventral oblique muscles. Scale bar: 7 μm. **d** Quantification. **e** Summary. **f** Reducing Nkx6 levels (*NB7–1-gal4 UAS-myr:GFP UAS-Nkx6-RNAi*) decreases projections out the ISNd to the ventral oblique muscles

We next asked whether Nkx6 was sufficient to generate VO motor neurons that correctly target the ventral oblique muscles. In wild type, NB7–1 always generated a motor neuron that exits the ISNd and targets ventral oblique muscles (Fig. 7a; volume of ISNd projections quantified in 7d). Overexpression of Nkx6 in the NB7–1 lineage generated additional innervation of the ISNd and ventral oblique muscles (Fig. 7b; quantified in Fig. 7d, S3). Importantly, the same phenotype was observed when individual neurons within the NB7–1 lineage are labeled by MCFO, showing that two distinct neurons in the lineage innervate ISNd (Fig. 7c), showing that at least one ectopic VO neuron can accurately project out the ISNd. These results are summarized in Fig. 7e. Conversely, when we reduced Nkx6 levels by RNAi (*NB7–1-gal4 UAS-myr:GFP UAS-Nkx6-RNAi*), we detected a loss of ISNd projections (Fig. 7f; compare to Fig. 7a). We conclude that Nkx6 is necessary and sufficient to generate VO motor neurons that project via ISNd to the ventral oblique muscles. Moreover, our data support a linear pathway in which the Kr/Pdm TTFs induce Nkx6 expression which specifies the molecular and morphological features of the VO motor neuron (see Discussion).

## Discussion

Kr/Pdm co-expression has been detected in several neuroblast lineages [6, 17], but until now there has not been evidence that this TTF combination could specify neuronal identity. We previously showed that the Kr/Pdm window generates a Kr/Pdm GMC [20] (Fig. S1), and here we show that this GMC generates an Nkx6^+^ ventral-projecting motor neuron. It is unknown whether Kr/Pdm directly or indirectly activate *nkx6* expression. The *nkx6* gene lies in a 45 kb region devoid of genes, and there are only a few, sparse predicted Kr or Pdm binding sites in this genomic expanse. How do Kr and Pdm together specify one fate (Nkx6^+^ VO motor neuron) whereas Kr or Pdm alone specify completely different fates (Eve^+^ dorsal motor neurons)? It is likely that Kr/Pdm together activate a different suite of target genes than either alone. For example, Kr/Pdm together may directly activate nkx6 expression, whereas neither alone has that potential. The emergence of single cell transcriptome and ChIP studies [32] will help to reveal how the combination of Kr/Pdm TTFs generates different cell fate output compared to Kr or Pdm alone.

The production of an Nkx6^+^ VO motor neuron in Kr/Pdm window interrupts the sequential production of Eve^+^ dorsal motor neurons in the NB7–1 lineage, resulting in an Eve>Nkx6 > Eve alternation of cardinal motor neuron production within the lineage. This is unusual, as in most cases neurons with similar morphology or function are produced together in a lineage. In mammals, progenitors generate neurons first, followed by glia [1]; we know of no examples of neuron>glia>neuron production from a single lineage. Similarly, *Drosophila* central brain neuroblast lineages produce the mushroom body γ neurons, then α’/ β’ neurons, and lastly α/β neurons, with no evidence for alternating or interspersed fates [33]. In the abdominal NB3–3 lineage, the early-born cells are in a mechanosensitive circuit, whereas the late-born cells are in a proprioceptive circuit [34]. To our knowledge, the only possible example of interleaved production of two morphological classes of neurons is in the *Drosophila* lateral antennal lobe neuroblast lineage, which alternate between uniglomerular and multiglomerular (AMMC) projection neurons [35]. The use of clonal and temporal labeling tools will be needed to examine additional lineages to determine the prevalence of lineages producing temporally interleaved neuronal subtypes as in the NB7–1 lineage.

Overexpression of Kr/Pdm or Nkx6 can induce only 2–3 ectopic VO motor neurons within the NB7–1 lineage. Clearly not all neurons in the lineage are competent to respond to these transcription factors. Early-born temporal identities specified by Hb and Kr (U1-U3) are unaffected by Kr/Pdm or Nkx6 overexpression, which is similar to previous data showing that early temporal fates are not affected by overexpression of later TTFs in multiple lineages [14, 28, 36]. It remains a puzzle why the Kr^+^ U3 neuron does not switch to a VO fate upon overexpression of Kr/Pdm. There may need to be an equal level of Kr and Pdm to specify VO fate, although this would not explain why Kr/Pdm overexpression converts the Pdm^+^ U4 motor neuron to a VO fate. Alternatively, there may be an early chromatin landscape that blocks access to relevant Pdm target loci.

We note that our assay of VO neuronal identity was done in newly-hatched larvae. Although motor circuits are functional at this time, larvae grow for five more days. We have no data on whether the ectopic VO motor neurons are functional or are maintained through the life of the larvae. This would be an important question for the future.

Nkx6 and Eve have cross-repressive interactions [14], but with some limitations: early-born Eve^+^ motor neurons are not affected by Nkx6 overexpression (our work and refs [14, 36]). Wild type animals even show sporadic expression of Nkx6 in the Eve^+^ U2 motor neuron (data not shown), but in these neurons it has no effect on Eve expression, nor does it promote targeting to ventral oblique muscles. There appears to be a mechanism to block endogenous or overexpressed Nkx6 function in the early lineage of neuroblasts producing Eve^+^ motor neurons. The mechanism “protecting” early-born Eve^+^ neurons from Nkx6 repression of Eve is unknown. Early lineages may lack an Nkx6 cofactor; Nkx6 could act indirectly via an intermediate transcription factor missing in early lineages; the early TTFs Hb or Kr may block Nkx6 function; or the *eve* locus could be in a subnuclear domain inaccessible to Nkx6.

Nkx6 promotes motor neuron specification in both *Drosophila* and vertebrates [13, 32]. In *Drosophila*, loss of Nkx6 reduces ventral projecting motor neuron numbers and increases the number of Eve^+^ neurons, while overexpression increases ventral projecting motor neuron numbers at the expense of Eve^+^ neurons (our work and [14]. In vertebrates the Nkx6 family members Nkx6.1/Nkx6.2 appear to play a broader role in motor neuron specification. Nkx6.1/Nkx6.2 show early expression throughout the pMN domain; mice mutant for both Nkx6 family members lack most somatic motor neurons; and Nkx6.1 overexpression in chick or zebrafish can induce ectopic motor neurons [36–38]. It would be interesting to investigate whether vertebrate Nkx6.1/Nkx6.2 are required to suppress a specific motor neuron identity, similar to the antagonistic relationship between Nkx6 and Eve in *Drosophila*.

Neuroblasts in all regions of the *Drosophila* CNS (brain, ventral nerve cord, optic lobe) use TTF cascades to generate neuronal diversity [3–6], yet less is known about TTF target genes. It is likely that TTFs induce expression of suites of transcription factors that persist in neurons and confer their identity. Examples may include the “morphology transcription factors” that specify adult leg motor neuron dendrite projections [39], but in this case it remains unknown whether these transcription factors control all other aspects of adult motor neuron identity. It is possible that “morphology transcription factors” are one module downstream of a broader regulatory tier similar to the terminal selector genes in *C. elegans* [13].

We identified a linear pathway from Kr/Pdm to Nkx6 which specifies VO motor neuron identity. TTFs could act by two non-mutually exclusive mechanisms: inducing a stable combinatorial codes of transcription factors that consolidate neuronal identity, or by altering the chromatin landscape to have a heritable, long lasting effect on motor neuron gene expression. Our observation that Nkx6 is maintained in the VO neuron after fading of Kr/Pdm expression (data not shown) supports the former mechanism. Identification of Kr/Pdm or Nkx6 target genes would give a more comprehensive understanding of TTF specification of neuronal identity.

The results presented in this work lead to several interesting directions. Other embryonic VNC lineages exhibit a Kr/Pdm window; does this window generate neurons in these lineages? Are there common features to neurons born in the Kr/Pdm window? Furthermore, do ectopic VO neurons make functional presynapses with the ventral oblique muscles, and do they have the normal inputs to their dendritic postsynapses? In only a few cases has it been shown the TTF-induced neurons are functionally integrated into the appropriate circuits [30]. Kr and Pdm orthologs have been identified in vertebrates. Looking for dual expression of Kr and Pdm orthologs in vertebrates may reveal a role in specifying temporal identity, similar to evidence for Hb and Cas TTFs having vertebrate orthologs that specify temporal identity [7, 9, 10, 40].

## Methods

### Fly stocks

Male and female *Drosophila melanogaster* were used. The chromosomes and insertion sites of transgenes (if known) are shown next to genotypes. Previously published gal4 lines, mutants and reporters used were: *NB7–1-gal4*^*KZ*^ (II) [21], called *NB7–1-gal4* here; *10XUAS-IVS-myr::sfGFP-THS-10xUAS(FRT.stop)myr::smGdP-HA* (RRID:BDSC_62127); *UAS-nkx6* (RRID:BDSC_9932); *UAS-nkx6*^*RNAi*^ (RRID:BDSC_61188); *UAS-Kr* (II and III) [15, 17]; *UAS-Pdm2* (II and III) [16, 28]; *hs-FLPG5.PEST.Opt* (RRID:BDSC_77140); *hs-FLPG5.PEST, 10xUAS(FRT.stop)myr::smGdP-OLLAS 10xUAS(FRT.stop)myr::smGdP-HA 10xUAS(FRT.stop)myr::smGdP-V5-THS-10xUAS(FRT.stop)myr::smGdP-FLAG* (RRID:BDSC_64086).

### Immunostaining and imaging

Primary antibodies were: mouse anti-Eve (5 μg/mL, DSHB, 2B8), rabbit anti-Eve #2472 (1:100, Doe lab), mouse anti-FasII (1:50, DSHB, 1D4), chicken anti-GFP (1:1000, RRID:AB_2307313, Aves Labs, Davis, CA), rabbit anti-HA epitope tag, DyLight™ 549 conjugated (1:100, Rockland, 600–442-384, Limerick, PA), rat anti-HA (1:100, MilliporeSigma, 11,867,423,001, St. Louis, MO), guinea pig anti-Hey (1:1000, gift from S. Bray, University of Cambridge), guinea pig anti-Kr (1:500, Doe lab), rat anti-Nkx6 (1:500, gift from J. Skeath, Washington University in St. Louis), rat anti-Pdm2 (abcam, ab201325, Cambridge, MA), guinea pig anti-Runt (1:1000, gift from C. Desplan, New York University), chicken anti-V5 (Bethyl Laboratories, Inc. A190-118A, Centennial, CO), rabbit anti-Zfh1 (1:1000, gift from R. Lehman, New York University), guinea pig anti-Zfh1 (1:1000, gift from J. Skeath, Washington University in St. Louis), and. Fluorophores-conjugated secondary antibodies were from Jackson ImmunoResearch (West Grove, PA) and were used at 1:200.

Embryos and the whole newly hatched larvae were fixed and stained as previously described [21]. In some cases, larval brains were dissected in PBS, fixed in 4% paraformaldehyde, and then stained by following protocols as described [21]. The samples were mounted either in Vectashield (Vector Laboratories, Burlingame, CA) or DPX [41].

Images were captured with a Zeiss LSM 710 or Zeiss LSM 800 confocal microscope with a *z*-resolution of 0.35 μm. Due to the complex 3-dimensional pattern of each marker assayed, we could not show NB7–1 progeny marker expression in a maximum intensity projection, because irrelevant neurons in the z-axis obscured the neurons of interest; thus, NB7–1 progeny were montaged from their unique z-axis position while preserving their X-Y position. This was done in Figs. 1, 4, and 6. Images were processed using the open-source software FIJI (https://fiji.sc). Figures were assembled in Adobe Illustrator (Adobe, San Jose, CA). Three dimensional reconstructions, and level adjustments were generated using Imaris (Bitplane, Zurich, Switzerland). Any level adjustment was applied to the entire image.

### Quantification of normalized ISNd volume

In order to generate a reliable volume metric for determining changes to the size of the ISNd nerve, FasII staining was used to identify SNc in a 3D IMARIS volume of the confocal image stack in order to normalize for variance in embryonic age and development. SNc was chosen to normalize against as we observed it to be unaffected by our genetic manipulations. Default parameters were used to generate a surface over the FasII channel labeling SNc and ISNd respectively, generating precise volume measurements. We then used these volume measurements to generate a ISNd/SNc ratio which was used for statistical analysis (see Fig. S1). This metric exhibited low variance across the wildtype controls, allowing for accurate comparison to genetically manipulated embryos.

### Statistics

Statistical significance is denoted by asterisks: ****p < 0.0001; ***p < 0.001; **p < 0.01; *p < 0.05; n.s., not significant. The following statistical tests were performed: Mann-Whitney U Test (non-normal distribution, non-parametric) (two-tailed *p* value) (Figs. 4c, f, i, j, 6D); Welch’s t-test (normal distribution, non-parametric) (two-tailed *p* value) (Figs. 5C, 7d). All analyses were performed using Prism8 (GraphPad). The results are stated as mean ± s.d., unless otherwise noted.

## Conclusions

We show that one neuroblast lineage generates interleaved cardinal motor neurons fates; that the Kr/Pdm TTFs form a novel temporal identity window that promotes expression of Nkx6; and that the Kr/Pdm > Nkx6 pathway is necessary and sufficient to specify VO motor neuron identity and morphology.

## Supplementary information

**Additional file 1: Figure S1.** NB7–1 sequentially expresses Kr, Kr/Pdm, and Pdm. NB7–1 is identified by expression of *NB7–1-Gal4 UAS-myr:GFP* (green) and Asense (Ase; blue). (A) At early stage 11, NB7–1 is Kr^**+**^ Pdm^**−**^. (B) At mid stage 11, NB7–1 is Kr^**+**^ Pdm^**+**^. (C) At late stage 11, NB7–1 is Kr^**−**^ Pdm^**+**^. Scale bar: 5 μm.

**Additional file 2: Figure S2.** Methodology for quantifying ISNd and SNc motor neuron localization. (A,A’) The volume of the ISNd was normalized to that of SNc to account for slight differences in embryo staging. ISNd and SNc were identified by the pan-motor axon marker FasII (magenta) in embryos expressing GFP (green) in the NB7–1 lineage (*NB7–1-gal4 UAS-GFP*). (B,B′) FasII (magenta) was used to identify SNc in a maximum intensity projection, and the volume quantified using the Imaris Surface function. (C,C′) FasII (magenta) was used to identify ISNd in a maximum intensity projection, and the volume quantified using the Imaris Surface function.

**Additional file 3: Figure S3.** Nkx6 induces ectopic VO motor neurons targeting ventral oblique muscles. (A) Control (*NB7–1-gal4 UAS-GFP*) shows innervation of the ISNd and ventral oblique muscles (arrowhead). (B) Overexpression of Kr and Pdm (*NB7–1-gal4 UAS-myr:GFP UAS-Kr UAS-Pdm*) lead to increased ISNd innervation (arrowhead). (C) Overexpression of Nkx6 (*NB7–1-gal4 UAS-myr:GFP UAS-Nkx6*) leads to excessive, broad, and disorganized innervation of ventral oblique muscles (arrowhead). (D) Nkx6 RNAi (*NB7–1-gal4 UAS-myr:GFP UAS-Nkx6-RNAi*) results in loss of ventral motor projections to ISNd (yellow arrow). Scale bar: 15 μm for all panels.

**Additional file 4: Supplemental Movie 1.** MCFO labels a single VO motor neuron within the NB7–1 lineage. (0:00–0:09) One hemisegment of a stage 16 embryo is shown in which the entire NB7–1 lineage is labelled (*NB7–1-gal4 UAS-GFP*, green**)**, and a single neuron from the lineage is sparsely labelled with an HA tag (red). Viewed together with FasII (blue) labelling nerve bundles, the axon of this morphologically-distinct motor neuron travels through the ISNd nerve route and terminates in a unique NMJ in the region of the ventral oblique muscles. Scale bar: 8 μm. (0:09–0:18) The VO motor neuron (HA, red), shown in relation to FasII (blue) possess a uniquely identifiable dendritic and axonal morphology.

## Data Availability

No datasets were generated. No new fly stocks were generated, and all fly stocks are available from public stock centers or by request.

## Abbreviations

Eve: Even-skipped
TTF: Temporal transcription factor
VO: Ventral oblique
Hb: Hunchback
Kr: Kruppel
Cas: Castor
NB7–1-gal4: NB7–1 split gal4
CNS: Central nervous system
OE: Overexpression
