# Supplementary figures and images for: A novel temporal identity window generates alternating Eve+/Nkx6+ motor neuron subtypes in a single progenitor lineage
Source: Neural Dev. 2020 Jul 28;15:9. doi: 10.1186/s13064-020-00146-6 (PMC7388218; doi:10.1186/s13064-020-00146-6)

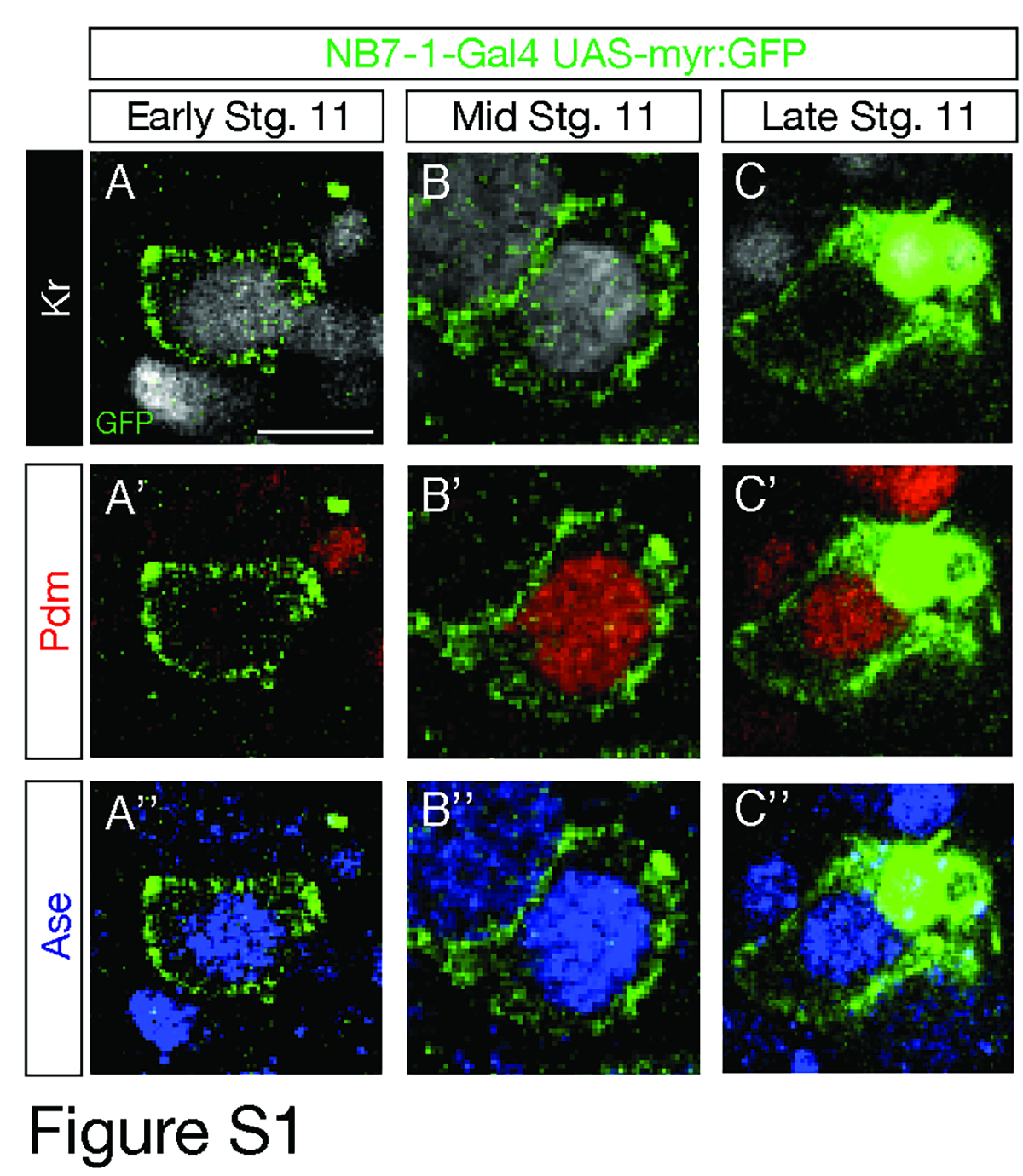

Supplement: Supplementary file 1 — Additional file 1: Figure S1. NB7–1 sequentially expresses Kr, Kr/Pdm, and Pdm. NB7–1 is identified by expression of NB7–1-Gal4 UAS-myr:GFP (green) and Asense (Ase; blue). (A) At early stage 11, NB7–1 is Kr+ Pdm−. (B) At mid stage 11, NB7–1 is Kr+ Pdm+. (C) At late stage 11, NB7–1 is Kr− Pdm+. Scale bar: 5 μm. [file 13064_2020_146_MOESM1_ESM.tif]

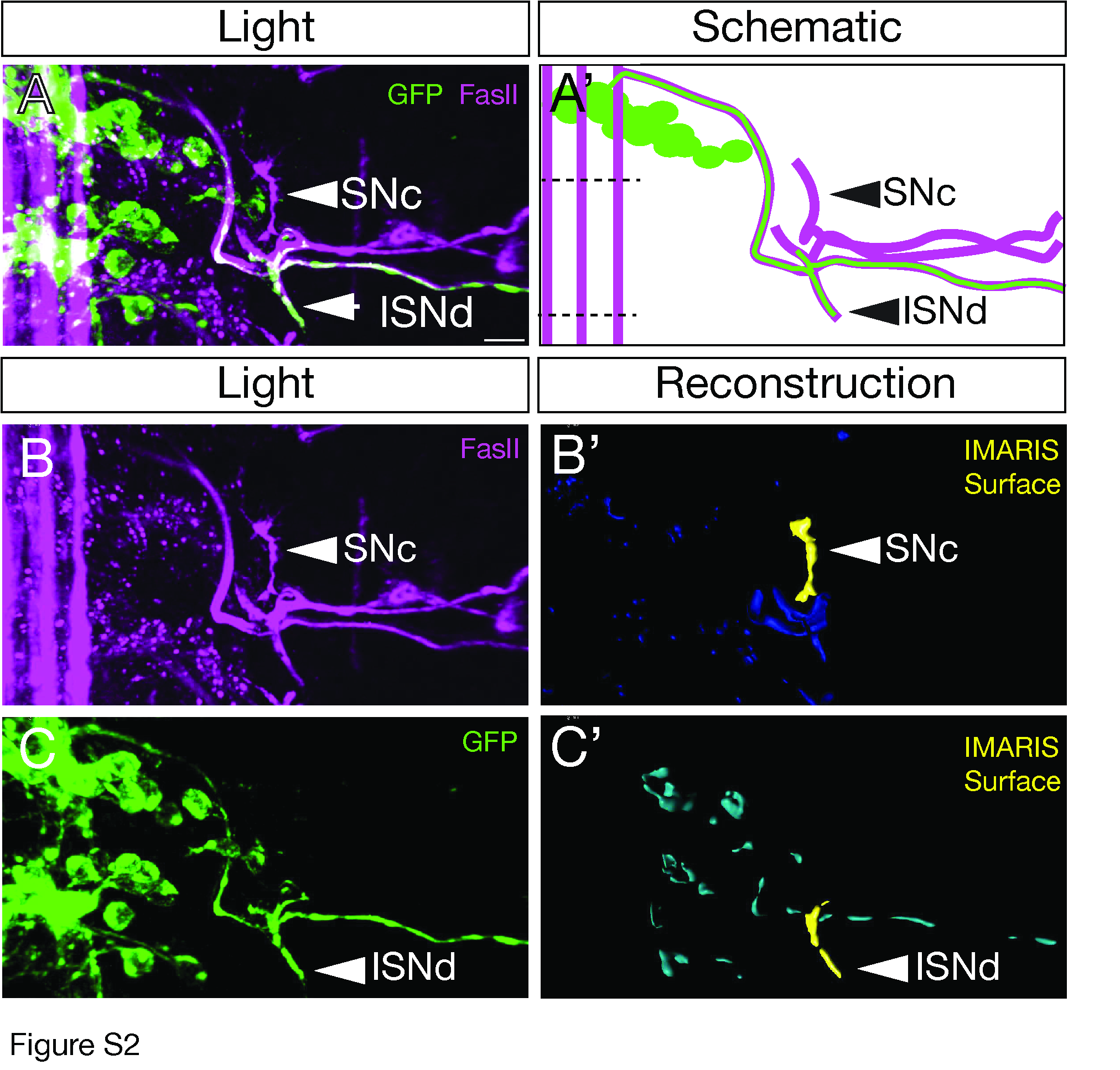

Supplement: Supplementary file 2 — Additional file 2: Figure S2. Methodology for quantifying ISNd and SNc motor neuron localization. (A,A’) The volume of the ISNd was normalized to that of SNc to account for slight differences in embryo staging. ISNd and SNc were identified by the pan-motor axon marker FasII (magenta) in embryos expressing GFP (green) in the NB7–1 lineage (NB7–1-gal4 UAS-GFP). (B,B′) FasII (magenta) was used to identify SNc in a maximum intensity projection, and the volume quantified using the Imaris Surface function. (C,C′) FasII (magenta) was used to identify ISNd in a maximum intensity projection, and the volume quantified using the Imaris Surface function. [file 13064_2020_146_MOESM2_ESM.tif]

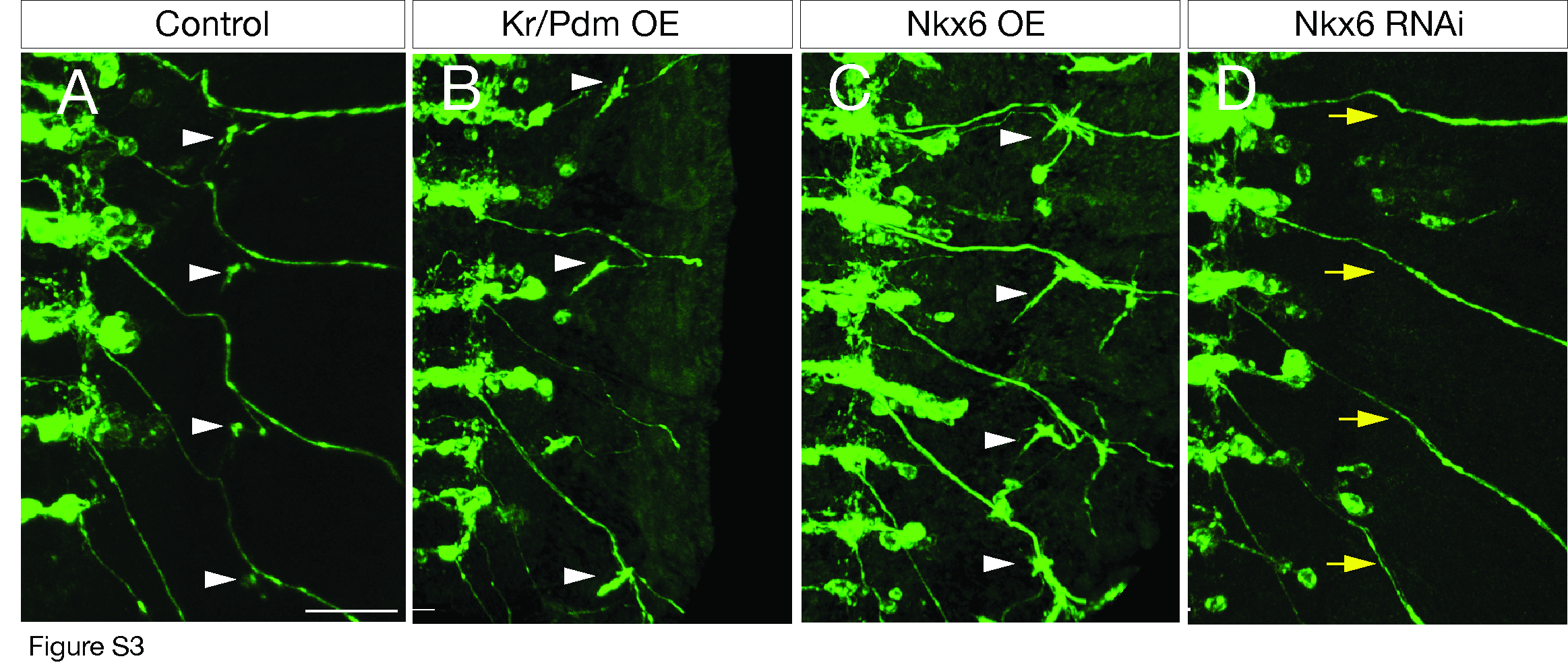

Supplement: Supplementary file 3 — Additional file 3: Figure S3. Nkx6 induces ectopic VO motor neurons targeting ventral oblique muscles. (A) Control (NB7–1-gal4 UAS-GFP) shows innervation of the ISNd and ventral oblique muscles (arrowhead). (B) Overexpression of Kr and Pdm (NB7–1-gal4 UAS-myr:GFP UAS-Kr UAS-Pdm) lead to increased ISNd innervation (arrowhead). (C) Overexpression of Nkx6 (NB7–1-gal4 UAS-myr:GFP UAS-Nkx6) leads to excessive, broad, and disorganized innervation of ventral oblique muscles (arrowhead). (D) Nkx6 RNAi (NB7–1-gal4 UAS-myr:GFP UAS-Nkx6-RNAi) results in loss of ventral motor projections to ISNd (yellow arrow). Scale bar: 15 μm for all panels. [file 13064_2020_146_MOESM3_ESM.tif]
